# Consequences of past and present harvest management in a declining flyway population of common eiders *Somateria mollissima*

**DOI:** 10.1002/ece3.5707

**Published:** 2019-10-02

**Authors:** Rune S. Tjørnløv, Roger Pradel, Rémi Choquet, Thomas Kjær Christensen, Morten Frederiksen

**Affiliations:** ^1^ Department of Bioscience Aarhus University Roskilde Denmark; ^2^ CEFE CNRS Univ Montpellier Univ Paul Valéry Montpellier 3 EPHE IRD Montpellier France; ^3^ Department of Bioscience Aarhus University Rønde Denmark

**Keywords:** CMR modeling, demography, hunting bag statistics, multi‐event model, population dynamics, prospective population scenario, sex ratio

## Abstract

1. Harvested species population dynamics are shaped by the relative contribution of natural and harvest mortality. Natural mortality is usually not under management control, so managers must continuously adjust harvest rates to prevent overexploitation. Ideally, this requires regular assessment of the contribution of harvest to total mortality and how this affects population dynamics.
2. To assess the impact of hunting mortality on the dynamics of the rapidly declining Baltic/Wadden Sea population of common eiders *Somateria mollissima*, we first estimated vital rates of ten study colonies over the period 1970–2015. By means of a multi‐event capture–recovery model, we then used the cause of death of recovered individuals to estimate proportions of adult females that died due to hunting or other causes. Finally, we adopted a stochastic matrix population modeling approach based on simulations to investigate the effect of past and present harvest regulations on changes in flyway population size and composition.
3. Results showed that even the complete ban on shooting females implemented in 2014 in Denmark, where most hunting takes place, was not enough to stop the population decline given current levels of natural female mortality. Despite continued hunting of males, our predictions suggest that the proportion of females will continue to decline unless natural mortality of the females is reduced.
4. Although levels of natural mortality must decrease to halt the decline of this population, we advocate that the current hunting ban on females is maintained while further investigations of factors causing increased levels of natural mortality among females are undertaken.
5. *Synthesis and applications*. At the flyway scale, continuous and accurate estimates of vital rates and the relative contribution of harvest versus other mortality causes are increasingly important as the population effect of adjusting harvest rates is most effectively evaluated within a model‐based adaptive management framework.

## Graphical abstract

We used multi‐event and stochastic population modeling to assess the past and present effect of hunting in a declining flyway population of common eiders. Our results indicate that even a ban on shooting female eiders is not enough to halt the current decline, because of high levels of natural mortality among the eider females.

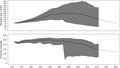

## 1 INTRODUCTION

Management of migratory bird populations requires detailed knowledge about their spatiotemporal dynamics in relation to resource availability and the interplay with human activities and interests affecting these relationships. In theory, flyway populations are spatially well‐defined biological units formed by groups of individuals with some degree of similarity in spatiotemporal movements, linked by common population processes through rates of immigration and emigration. As a result, flyways have become the primary unit of focus for conservation and management of migratory birds (Boere, Galbraith, & Stroud, 2006). Effective flyway population management necessitates an understanding of population delineation and knowledge of connectivity, gene flow, location of protected areas, and environmental and anthropogenic pressures throughout the annual cycle (Madsen, Tjørnløv, Frederiksen, Mitchell, & Sigfusson, 2014). Flyway management also requires good knowledge of total population size and its rate of change over time. Harvest in wintering areas or along migration routes can potentially affect the rate of change of flyway populations through elevated rates of mortality. Within Europe, the high annual kill relative to estimated population size in some populations has led to concern about the contribution of hunting to observed declines (Hirschfeld & Heyd, 2005). Recent studies on the effects of hunting of popular quarry species (including waterbirds) on their population trends have assessed the covariance between time series of population trend estimates and various measures of hunting pressure (i.e., the number of animals shot relative to population size) or compared population trends of hunted versus nonhunted species (Jiguet, Godet, & Devictor, 2012; Pöysä, Rintala, Lehikoinen, & Väisänen, 2013). However, the extent to which harvest affects the population dynamics of European waterbirds through elevated mortality remains poorly understood (Devineau, Guillemain, Johnson, & Lebreton, 2010). Consequently, the need to extend current monitoring through extensive and long‐term marking projects of quarry species to facilitate formal estimation of survival and mortality rates due to hunting has recently been highlighted (Elmberg et al., 2006). In addition, assessments of a specific management action on the dynamics of a flyway population must consider the possibility of diverging population processes occurring within the same population. Ideally, understanding these processes requires inclusion of data from several representative study sites to effectively scale up and model effects of management at the flyway level (Grosbois et al., 2009).

The common eider *Somateria mollissima* (hereafter eider) is a large seaduck and a popular quarry species along several of its circumpolar flyways, for instance, in Greenland and Canada and in northwest Europe (Christensen & Hounisen, 2014; Gilliland et al., 2009). In contrast, populations in the UK and Iceland are fully protected (Waltho & Coulson, 2015). Several hunted flyway populations of eiders show declining population trends raising the question of how recreational harvest and subsistence harvest contribute to current population declines. For example, Gilliland et al. (2009) estimated that harvest rates on Greenlandic wintering quarters were highly unsustainable and advised major harvest reductions which helped the population to recover (Merkel, 2010). The Baltic/Wadden Sea flyway population of eiders has also suffered a major decline of an estimated 36% between 1991 and 2000 (Desholm et al., 2002). The breeding range of this population extends from the northern Baltic Sea, through the coasts of Sweden and Denmark to the Wadden Sea in The Netherlands, whereas the main wintering ground, which is shared among the different population divisions, is located in the Wadden Sea and in inner Danish waters. Among the countries sharing this population, eider hunting is most extensive in Denmark. Under the provisions of the EU Bird Directive, Habitat Directive, and Ramsar, Bern, and Bonn conventions, all member states have a special responsibility to ensure that eider hunting within their jurisdiction is sustainable. In light of recent flyway population declines, there is thus an urgent need to assess the population consequences of current levels of hunting. After more than a decade of declines, multiple adjustments to sex‐specific hunting season lengths were implemented in Denmark to counteract this negative population trend (Christensen & Hounisen, 2014). This resulted in a drop of 82% in the bag of adult females and 31% and 58% in first‐winter females and males, respectively. Despite this large reduction of the harvest, a complete ban on hunting females was implemented in Denmark in 2014 to be evaluated in 2018 (Asferg et al., 2016). Using baseline demographic data from a single Danish eider colony, Christensen and Hounisen (2014) predicted that a ban on hunting female eiders would ultimately result in an annual population growth of 0.7%. However, to thoroughly evaluate the effect of hunting regulations on flyway survival rates, it is essential to capture potential differences in the contributions of hunting and natural mortality affecting different divisions of the same flyway population.

In this analysis, we attempt to advance our knowledge of the Baltic/Wadden Sea eider population in order to support sustainable harvest management. As a basis for our investigation, we used vital rates estimated from modeling of capture–recapture–recovery data from several study sites. We then used the reported cause of death associated with an extensive number of recoveries to estimate proportions of birds dying due to hunting and other causes. By allowing for adjustments in survival rates depending on hunting intensity, we explored the flyway population consequences of past and present harvest management using a stochastic demographic model. We hypothesize that the efficacy of using hunting regulations to increase flyway population size largely depends on current levels of natural mortality.

## 2 MATERIALS AND METHODS

### 2.1 Species and study area

The eider is a colonially breeding seaduck, with a circumpolar distribution comprising several discrete flyway populations (Goudie, Robertson, & Reed, 2000). One such population extends from the upper Baltic Sea to the Dutch Wadden Sea, involving both fully and partially migrant subpopulations (Ekroos, Fox, et al., 2012). More than half of the total flyway population overwinters in Danish waters (Ekroos, Fox, et al., 2012). Between the early 1960s and mid‐1990s, the annual Danish bag ranged 100,000–200,000 individuals corresponding to 6.5%–9.5% of the flyway population (Noer, Clausager, & Asferg, 1995). Over the same period of time, annual bags in Finland and Sweden ranged 25,000–27,000 and 3,000–5,000 birds, respectively (Desholm et al., 2002).

Recently, annual bag sizes have fallen to 40,000–60,000, 4,000–6,000, and 5,000 birds in Denmark, Finland, and Sweden, respectively (Christensen & Hounisen, 2014). The reduction in Denmark was partly due to falling popularity of seaduck hunting (Christensen, 2005) and partly to multiple sex‐specific reductions in the length of the hunting season of males and females by one and three months, respectively, followed by a complete female hunting ban from the hunting season 2014/2015 (Christensen & Hounisen, 2014).

### 2.2 Data

**Figure 1:**
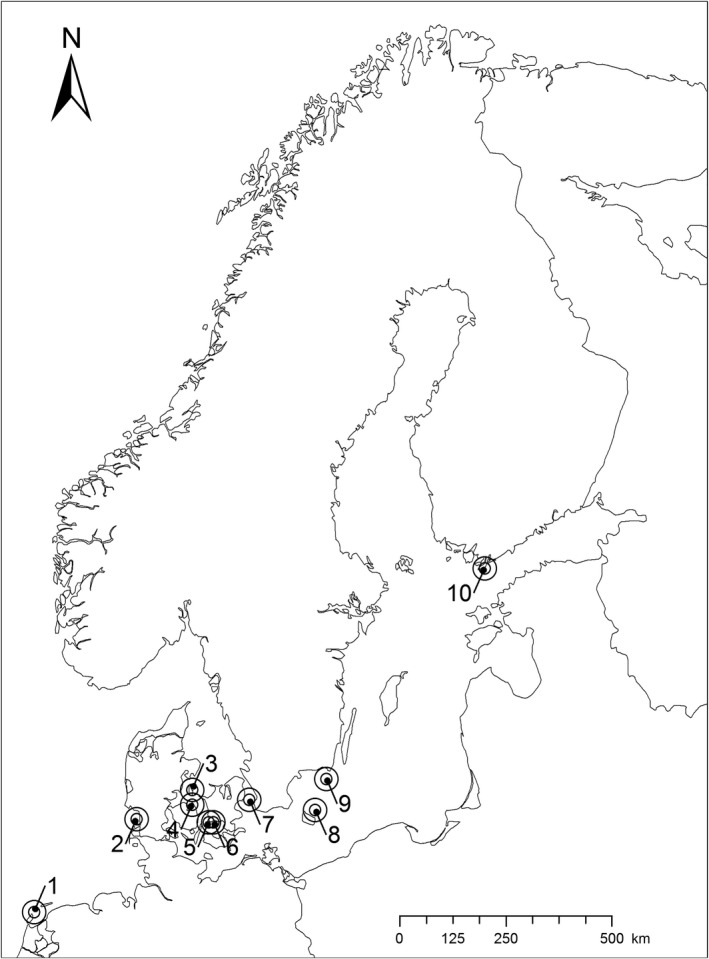
Sites with available long‐term capture‐recapture‐recovery data: 1: Vlieland (NL); 2: Mandø (DK); 3: Stavns Fjord (DK); 4: Hindsholm (DK); 5: Helleholm, Agersø (DK); 6: Næbbet, Stigsnæs (DK); 7: Saltholm (DK); 8: Christiansø (DK); 9: Utklippan (S); and 10: Tvärminne (FIN)

In total, we used encounter histories of some 22,300 common eider females ringed at ten study sites (Figure 1) and 1,300 adult males ringed at Vlieland (study site 1) in the Dutch Wadden Sea which was the only colony with data available on males. Data of some 5,600 ducklings were compiled from study site 8, Christiansø (for more details of the data, see Appendix).

### 2.3 Statistical analysis

#### 2.3.1 Step 1—Flyway vital rates

Grouped by site, 46 years (1970–2015) of ten colony‐specific mark–recapture–recovery datasets of breeding females were arranged in one encounter history file. We used the Burnham model (Burnham, 1993) for mixtures of live‐and‐dead encounter data implemented in Program MARK (White & Burnham, 1999). The Burnham model estimates survival (*S*) unbiased by permanent emigration, recapture probability (*p*), recovery probability (*r*), and site fidelity (*F*) (Burnham, 1993). We specified models with time‐dependent and site‐specific survival and recapture probabilities, while using Akaike's information criterion adjusted for small sample sizes (AIC_c_) to select between candidate models varying with respect to recovery and site fidelity (Burnham & Anderson, 2002).

Annual survival of prebreeders and adult males was estimated for colonies with suitable data. For prebreeders, we structured these models with three age classes for survival, four age classes for recapture, and two age classes for recovery and site fidelity.

#### 2.3.2 Step 2—Hunting mortality of adult females

For this step, we used only dead recoveries and pooled data from all colonies. Following Schaub and Pradel (2004) and Souchay and Schaub (2016), we used the known cause of death reported by the public to tease apart mortality due to hunting (_h_) versus other (_o_) causes. We used program E‐SURGE (Choquet, Rouan, & Pradel, 2009) to develop a multi‐event ring‐recovery model that simultaneously estimated survival (*S*), proportions of birds dying due to either cause (*α*_h,_ *α*_o_), cause‐specific recovery probabilities (*r*_h,_ *r*_o_), and probabilities that dead individuals were assigned to the correct cause (*δ*_h_, *δ*_o_). We specified a model with four biological states: alive, newly dead due to hunting, newly dead due to other causes, and old dead. The newly dead states ensured that encounters of dead birds were assigned to only one recovery period, whereas the old dead state was absorbing and unobservable (Gauthier & Lebreton, 2008). Conditional on the states, we specified an observation process with four events: not seen, recovered dead due to hunting, recovered dead due to other causes, and recovered dead with no information of the cause (see Appendix for state and event matrices and more details of the model). We assumed equal mortality rates due to hunting across colonies because birds within the flyway mix during the hunting season. As we experienced that not all parameters of the global model (time dependence for all parameters) were separately identifiable, we specified simpler candidate models. We first specified models with one or both of the cause‐specific recovery probabilities (*r*_h,_ *r*_o_) held constant over time. Next, we constrained the recovery probability due to other causes with a linear trend because evidence suggests it may have declined over time in northwest Europe (Frederiksen & Bregnballe, 2000; Robinson, Grantham, & Clark, 2009). For a more robust likelihood optimization procedure, we used the option “multiple random initial values” over successive runs.

To validate the results of the multi‐event model, we computed the annual killrate, that is, the probability that an adult female eider alive at occasion *i* dies due to hunting between occasion *i* and occasion *i* + 1:

(1) $${\text{killrate}}_{h,\;\text{ad}\;\text{female},\;i}=\alpha_{h,\;\text{ad}\;\text{female},\;i}*\left[1-S_{\text{ad}\;\text{female},\;i}\right]$$

and compared this to a similar probability, that is, a hunting pressure (HP) calculated based on The Danish Hunting Bag Record (Strandgaard & Asferg, 1980) and The Danish Wing Survey (see Clausager (2004) and references therein, http://www.bios.au.dk/vinger):

(2) $${\text{HP}}_{\text{age}-\text{sex},\;i}=\text{no}{\text{. bagged}}_{i}*\frac{\text{no}{\text{. wings}}_{\text{age}-\text{sex},\;i}^{\text{shot}}}{\sum \text{no}{\text{. wings}}_{i}^{\text{shot}}}/N_{\text{age}-\text{sex},\;i}^{\text{postbreed}}$$

where no. bagged*_i_* is the total number of birds bagged in year *i*, no. wings_age_ *_–_*_sex,_ *_i_* is the number of collected wings of shot birds in each age–sex class in year *i*, Σno. wings*_i_* is the sum of all wings collected in year *i*, and $N_{\text{age/sex},i}^{\text{postbreeding}}$ is the number of birds in each age–sex class present in the population immediately following the breeding season (from a postbreeding version of the population model described below). Because of data limitations, proportions of immatures and males that died due to hunting could not be directly estimated within the multi‐event model as for the adult females. Because the killrate_h_ (Equation 1) and the HP (Equation 2) both express the probability that an eider died due to hunting between year *i* and year *i +* 1, we substituted killrate_h, immatures–males,_ *_i_* with HP_immatures–males,_ *_i_* (Equation 1) and calculated the lacking proportions of immatures and males that died due to hunting.

#### 2.3.3 Goodness‐of‐fit testing

Prior to model fitting, we assessed the degree of overdispersion. Because the currently available goodness‐of‐fit test for multistate models (Pradel, Wintrebert, & Gimenez, 2003) does not allow for unobservable states, this method was considered inappropriate. We therefore computed the median c‐hat in Program MARK for an analogous capture–recovery model which did not consider separate causes of death. The resulting median c‐hat value can thus be considered conservative in relation to both step 1 and step 2 because these models decrease heterogeneity by splitting data by colony (step 1) or mortality into two components (step 2).

#### 2.3.4 Overview of the population model

**Figure 2:**
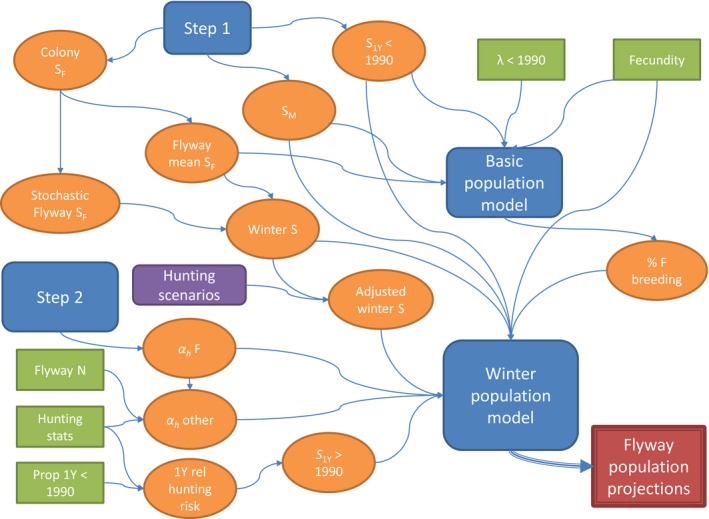
Conceptual diagram showing the flow of data, analyses, and modeling involved in projections of flyway population change. Blue elements indicate the main steps of the analysis, green elements indicate external information (literature values and hunting stats), orange elements indicate estimated quantities, purple elements indicate hypothetical scenarios, and dark red indicates the main aim of the analysis: projections of flyway population size and composition

Figure 2 provides an overview of the complex process involved in modeling of the flyway population of eiders and population consequences of prevailing hunting restrictions.

Not all demographic quantities necessary to model the population were directly available, and some parameters had to be approximated while making assumptions and by including other data sources.

By assuming a population growth rate of 3.46% prior to 1991 as indicated by aerial surveys (Joensen, 1974), we parameterized a basic population projection model with survival rates from step 1 and age‐specific fecundity (clutch size × hatching success) of 4.1 (Christensen & Hounisen, 2014) in order to approximate the probability of breeding after the second year of life, that is, for adults (*b*_3_). Initial population sizes (i.e., in 1970) were back‐calculated based on the assumed population growth rate prior to 1991 and a flyway population size in 1991 of 1.7 million birds (Rose & Scott, 1997):

(3) $${\text{pop}}_{1970\;n1-6}=\left(\frac{{\text{pop}}_{1991}}{\lambda^{21}}\right)*{\text{age}}_{\text{dist}\;n1-6}$$

where pop_1970_ *_n_*_1–6_ is the population size in 1970 of the six age and sex classes, pop_1991_ is the flyway population size in 1991, *λ* is the population growth prior to 1991, and age_dist_ *_n_*_1–6_ is the stable age distribution originating from the basic population model. Given the assumption that no breeding occurs before the third year of age (*b*_2_ = 0), we then estimated the probability of breeding among third‐year or older individuals (*b*_3_) by adjusting this parameter to match the assumed population growth of 3.46%.

An estimate of first‐year survival was only available for the initial period of population increase, up to 1981. To approximate survival from the subsequent period, we used data from The Danish Hunting Bag Record and The Danish Wing Survey, which annually since 1982 has assigned age and sex of 1.3%–4.0% of all eiders shot in Danish waters. Assuming that first‐year birds constituted c. 28% of the total population at the start of the open season in the early 1990s (Noer et al., 1995) and that the relative hunting risk of first‐year individuals was the same before and after the decline (but prior to the first sex‐differentiated hunting regulation), we calculated the proportion of first‐year (1Y) birds for the phase of decline:

(4) $$\begin{array}{c} \hat{{\text{prop1Y}}_{\text{decline}}^{\text{pop}}}=\frac{\bar{{\text{prop1Y}}_{\text{decline}}^{\text{bag}}}}{\hat{\text{Relrisk}}}\\ \hat{\text{Relrisk}}\;\text{= logit}\left(\bar{{\text{prop1Y}}_{\text{increase}}^{\text{bag}}}\right)-\text{logit}\left(\bar{{\text{prop1Y}}_{\text{increase}}^{\text{pop}}}\right) \end{array}$$

where $\hat{\text{Relrisk}}$ is the estimated relative hunting risk of first‐winter birds and logit(*p*) = ln(*p*/(1 − *p*)).

By trial and error, we then used a population projection model (presented in detail below) to adjust first‐year survival in order to match the decline in percentage among first‐winter birds at the start of the hunting season.

#### 2.3.5 A population model with a midwinter census

Waterbirds are usually counted during winter (Scott & Rose, 1996), when birds from multiple colonies assemble in large flocks, which makes them easier to count. To be able to compare empirical monitoring data, that is, population counts, with our model predictions, we constructed a Lefkovitch transition matrix that tracks the population at midwinter. Our age‐specific estimates of survival (breeding‐to‐breeding) therefore had to be adjusted to fit with a midwinter population census. We assumed that the elevated mortality during the first year of life was restricted to the period from hatching to midwinter and that survival during the second half of the first year of life was comparable to the second year of life. Denoting our mean estimates of survival $S_{0}^{\text{step}1}$, $S_{1}^{\text{step}1}$, $S_{\text{ad}\;\text{female}}^{\text{step}1}$, and $S_{\text{ad}\;\text{male}}^{\text{step1}}$, we computed the relevant parameters for the matrix model as:

(5) $${\text{SW}}_{0}=\frac{S_{0}^{\text{step}1}}{\sqrt{S_{1}^{\text{step}1}}}$$

(6) $${\text{SW}}_{1}=S_{1}^{\text{step1}}$$

(7) $${\text{SW}}_{2}=\sqrt{S_{1}^{\text{step1}}}*\sqrt{S_{\text{ad}\;\text{female}}^{\text{step1}}}$$

(8) $${\text{SW}}_{\text{adF}}=S_{\text{ad}\;\;\text{female}}^{\text{step1}}$$

(9) $${\text{SW}}_{\text{adM}}=S_{\text{ad}\;\;\text{male}}^{\text{step1}}$$

#### 2.3.6 The midwinter–midwinter matrix model

$$\left[\begin{array}{cccccc} 0 & f_{2}F & f_{\text{ad}}F & 0 & 0 & 0\\ {\text{SW}}_{1}F & 0 & 0 & 0 & 0 & 0\\ 0 & {\text{SW}}_{2}F & {\text{SW}}_{\text{ad}}F & 0 & 0 & 0\\ 0 & f_{2}M & f_{\text{ad}}M & 0 & 0 & 0\\ 0 & 0 & 0 & {\text{SW}}_{1}M & 0 & 0\\ 0 & 0 & 0 & 0 & {\text{SW}}_{2}M & {\text{SW}}_{\text{ad}}M \end{array}\right]\left[\begin{array}{c} n_{1}F\\ n_{2}F\\ n_{\text{ad}}F\\ n_{1}M\\ n_{2}M\\ n_{\text{ad}}M \end{array}\right]$$

The midwinter matrix population model had three age classes: first‐winter (_1_), second‐winter (_2_), and adult (_ad_); and two sex classes: females (F) and males (M), allowing simultaneous monitoring of the dynamics of the population sex ratio. Fertility rates (*f*) expressed as the number of first‐winter females (*f*_2_F + *f*_ad_F) and males (*f*_2_M + *f*_ad_M) produced per second‐winter (_2_F) and adult winter female (_ad_F) were approximated as:

(10) $$f_{2}F=f_{2}M=\sigma *z*\sqrt{S_{1}F}*b_{2}*{\text{SW}}_{0}$$

(11) $$f_{\text{ad}}F=f_{\text{ad}}M=\sigma *z*\sqrt{S_{\text{ad}}F}*b_{3}*{\text{SW}}_{0}$$

where *σ* denotes the primary sex ratio, that is, the sex distribution at birth (assumed to be 0.5); *z* is the fecundity, that is, the number of hatchlings per breeding female; and *b*_2_ and *b*_3_ are age‐specific breeding probabilities. Values of fecundity were derived from the literature (Bregnballe, 1991).

Based on these demographic quantities (see Appendix for an overview), we constructed a midwinter‐to‐midwinter, time‐dependent matrix population model. We then included among‐colony variation and stochasticity in adult female survival (*S*_ad_F) and clutch size by means of simulations that for every iteration (*n* = 10,000) and every year (*n* = 45) randomly drew a new estimate from a density distribution function given by the mean and standard error of the estimates while the population was projected over time. Likewise, stochasticity was also included for the other survival parameters.

Scenarios reflecting changes in hunting intensity were generated by adjusting reference survival rates ultimately resulting in changes of the matrix elements in the population model. We used the time frame of prevailing hunting regulations to extract mean survival rates and adjusted these according to changes in hunting intensity (corrected from Schaub & Pradel, 2004):

(12) $$S_{\text{adj,}\;\;i}=1-\left(1-S_{\text{base}}\right)*\left(1-\alpha_{h,\;\;\text{base}}\right)-\left(1-c*x_{i}\right)*\left(1-S_{\text{base}}\right)*\alpha_{h,\;\text{base}}$$

where *S*_base_ and *α*_h, base _are mean survival and proportion of birds dying due to hunting during the reference period, *c* is the degree of compensation (fully additive effect = 1, fully compensatory effect = 0), *x_i_* is the % change in *α*_h_ relative to the reference, and *S*_adj_ is then the adjusted survival rate given the change in hunting intensity. Given the long life expectancy of the eider and because we track the population from the time of midwinter where natural mortality is no longer high for first‐year birds, we assumed a fully additive effect of hunting (*c* = 1).

Given the estimated and approximated demographic rates, we projected the Baltic/Wadden Sea eider population between 1970 and 2014. Subsequently, we used survival rates adjusted for changes in hunting intensity following the most recent hunting regulations to predict the prospective population trajectory between 2014 and 2025.

## 3 RESULTS

Goodness of fit of a capture–recovery model with no stratification of mortality, time‐dependent survival, and a trend on the recovery rate indicated a slight lack of fit (median c‐hat ± 95% CI = 1.60 ± 0.17). Therefore, we did not expect bias in parameter estimates of either the colony‐specific survival analysis (step 1) or the cause‐specific mortality analysis (step 2) that stratifies mortality based on the known cause of death.

Assuming common recovery rates (*r*), a subset of candidate models estimated time series of colony‐specific adult female survival (step 1, estimates presented in Appendix) over 45 years (1971–2015). QAIC model selection identified a model with a common and linear trend over time (*T*) on recovery rates (except Vlieland) as the most parsimonious: *S*(g*t)* p*(g*t)* r*(*T*, VL)* F*(*g*). In addition, separate single‐site capture–recapture models also estimated first‐year survival of hatchlings $S_{0}^{\text{step}1}$ = 0.12, second‐year survival $S_{1}^{\text{step}1}$ = 0.83, and adult male survival $S_{\text{adM}}^{\text{step1}}$ = 0.92.

Among the set of candidate models, some of the cause‐specific (multi‐event) mortality models (step 2) were overparameterized and generated unreliable parameter estimates with large standard errors. However, a constraint on recovery rates from causes other than hunting (representing a decline over time as in step 1) facilitated identifiability of all parameters. In step 2, model *S*(*t*),*α*_h_(*t*),*r*_h_,*r*_o_(*T*),*δ*_h_,*δ*_o_ was selected as the best (deviance: 34,049, no. of parameters: 95), clearly better than the second best model candidate *S*(*t*),*α*_h_(*t*),*r*_h_,*r*_o_,*δ*_h_,*δ*_o_ (deviance: 34,144, no. of parameters: 94, delta AIC: 93). According to the best model, the probability for hunters to recover rings of shot birds was estimated as 0.36 (95% CI, 0.25–0.49), whereas the recovery probability of birds dead due to other causes fell linearly from 0.32 to 0.04 between 1970 and 2015; similar declines have been reported for other species (Frederiksen, Korner‐Nievergelt, Marion, & Bregnballe, 2018; Robinson et al., 2009). Furthermore, the probability that recoveries were correctly assigned to hunting (*δ*_h_) and other causes (*δ*_o_) was estimated as 0.94 and 0.95, respectively.

**Figure 3:**
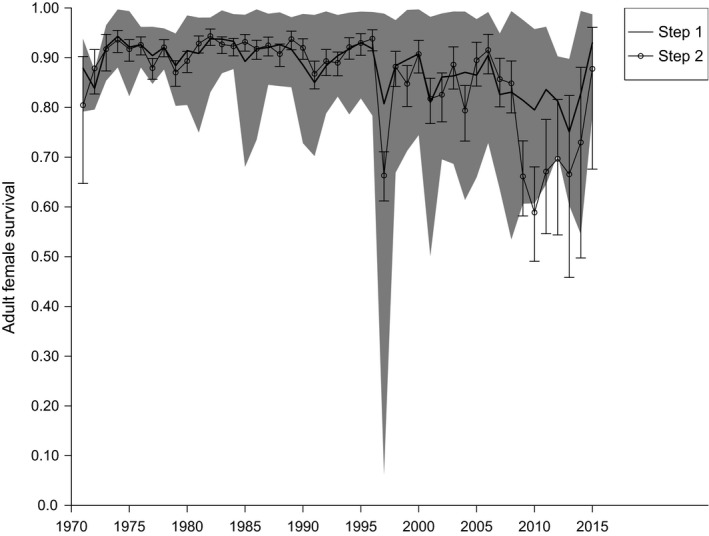
Estimates of adult female survival at the flyway level from a colony‐specific capture–recapture–recovery analysis (step 1), and a multi‐event model with two causes of death, that is, hunting and other causes (step 2). The black solid line and gray‐scaled areas represent mean estimates of simulations based on step 1 and 95% confidence intervals, respectively. Connected open circles and error bars represent estimates and 95% confidence intervals from step 2

Estimates of adult female survival from steps 1 and 2 were high and stable as the population grew until the early 1990s, but declined markedly hereafter, in particular after the mid‐1990s (Figure 3).

It is evident that the cause‐specific multi‐event model applied in step 2 tended to underestimate survival relative to step 1, especially over the most recent period. Because live recaptures were included in step 1 and not in step 2, we considered survival estimates from step 1 more accurate and used these to model the flyway population of eiders. Mean adult female survival in the nonhunted colony of Vlieland was 0.90, only 1% point higher than in the other study colonies without incidence of epidemic disease.

**Figure 4:**
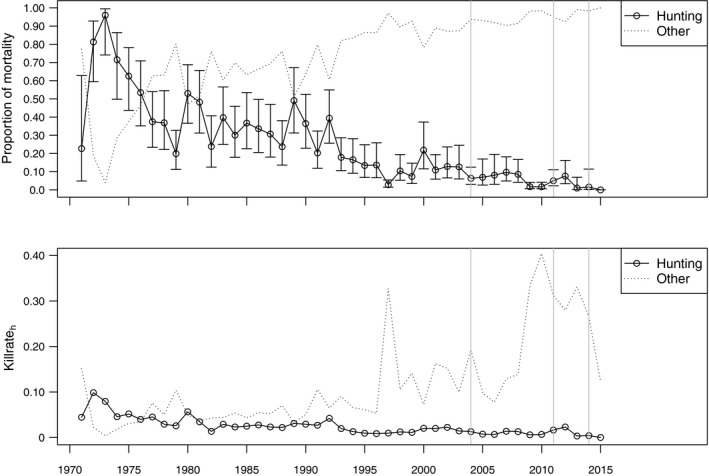
Upper panel shows estimates from step 2; the proportion of mortality of adult females caused by hunting (open connected circles) and other causes (dotted line) between 1971 and 2015. Error bars on the proportion dying due to hunting indicate 95% confidence intervals. Lower panel shows the resulting killrate, which is the probability of dying due to hunting or other causes (lower panel). Gray, vertical lines indicate the implementation of past hunting regulations

Significant declines in both *α*_h_ (*r* = −.83, *n* = 45, *p* < .01) and killrate_h_ (*r* = −.77, *n* = 45, *p* < .01) indicated major reductions in the hunting pressure over time (Figure 4).

**Figure 5:**
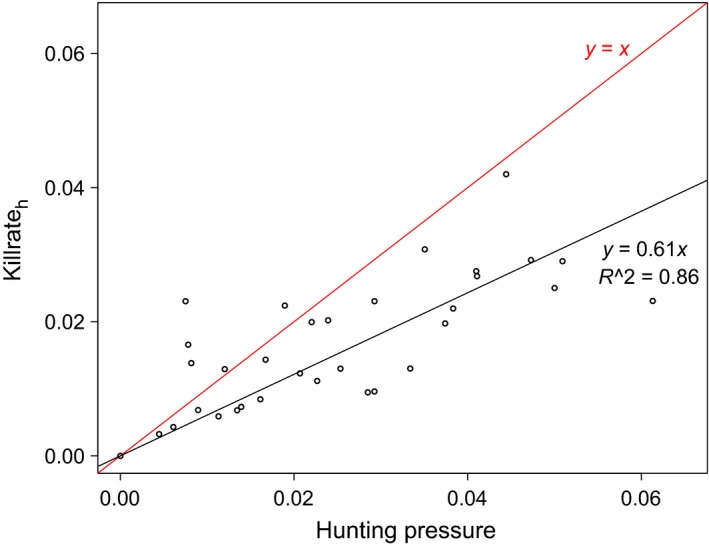
Relationship between the hunting pressure based on bag statistics and the killrate from hunting derived from results of a multi‐event model with two causes of death, that is, hunting and other causes (step 2). The red line indicates a 1:1 relationship

The two independent measures of hunting intensity were highly correlated, but the killrate_h_ from the analysis in step 2 was consistently lower than the analogous hunting pressure calculated from bag statistics (Killrate_adult female_ = 0.61 × HP_adult female_, *R* ^2^ = 0.86; Figure 5).

By means of a basic population model and a target population growth rate, the probability of breeding among adults (*b*_3_) was by trial and error estimated as 0.70. The proportion of first‐year individuals in the hunting record and their relative hunting risk indicated that survival of hatchlings had declined to 0.09 (0.12 prior to the early 1990s).

**Figure 6:**
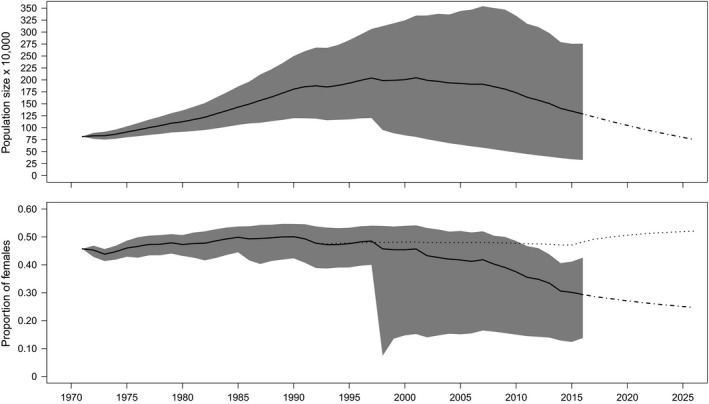
Population trajectory of the midwintering Baltic/Wadden Sea flyway population of eiders between 1971 and 2025 (upper panel). The black solid and dot‐dashed lines represent mean retrospective and prospective population trajectories, respectively. The lower panel shows the population sex ratio as the proportion of females among adult birds. Black solid and dot‐dashed lines are under the assumption of constant male survival, whereas the black dotted line assumes a similar absolute change in adult male survival since 1991 (on the logit scale) as observed for the females. Gray‐scaled areas indicate 95% confidence intervals, which originate from simulations based on variation between study colonies

The reconstructed population trajectory suggested a relatively weak population increase of 8% throughout the 1990s, followed by a considerable decline in population size of 35% between years 2000 and 2014 (Figure 6, upper panel). Given high rates of natural mortality, the recently implemented hunting ban on females appears insufficient to fully counteract the observed decline. The scenario with stable survival of adult males resulted in annual growth of 0.95 for the interval 2014–2025; hence, natural mortality must decrease in order to revert this negative population trend (Figure 6, upper panel). We further predict that despite the current hunting ban on females, the proportion of females in the population will continue to decrease from 30% in 2014 (among adults) to 25% in 2025 (Figure 6, lower panel).

## 4 DISCUSSION

### 4.1 Population trends and processes

Despite multiple restrictive hunting regulations, our reconstructed population trajectories indicate that the wintering Baltic/Wadden Sea flyway population of eiders was reduced considerably since year 2000. Moreover, model predictions indicate that levels of natural mortality among adult females have recently increased to an extent that cannot be counteracted through hunting regulations. Population trends of the Baltic/Wadden Sea flyway population differ according to source (Ekroos, Fox, et al., 2012). While midwinter counts suggest a significant population reduction throughout the 1990s (followed by a slight increase), nest counts indicate a considerable population decline between 2000 and 2009. It has been suggested that this discrepancy arises from a delay in age of first breeding, an increase in nonbreeding frequency, changes in census methods, or combinations of these factors (Ekroos, Fox, et al., 2012). Based on demographic data from multiple colonies, we present a reconstructed population trajectory that seems to confirm the trend inferred from nest counts, that is, that the largest population reduction occurred after year 2000. It is important to note that our results rely on the assumption that our study colonies are representative of the overall population.

Factors causing increased natural mortality rates among breeding females likely differ between population divisions. For example, in the Western Gulf of Finland incubating females suffer from predation by a rapidly growing population of white‐tailed sea eagles *Haliaeetus albicilla* and non‐native American mink *Neovison vison* (Ekroos, Öst, Karell, Jaatinen, & Kilpi, 2012). Outbreaks of avian cholera have struck several eider colonies in The Netherlands, Denmark, and Sweden, in some cases wiping out 90% of the females nesting there (Christensen, Bregnballe, Andersen, & Dietz, 1997; Swennen & Smit, 1991; Tjørnløv, Humaidan, & Frederiksen, 2013). Moreover, an unusually large number of eiders have been found dead at Christiansø in the Baltic Sea, presumably due to starvation at the end of the breeding season (Garbus et al., 2018). Food deficiency, associated with lower nutrient levels in the marine environment (Laursen & Møller, 2014) and overharvest of shellfish (Camphuysen et al., 2002), may also affect body condition as prebreeding females rely on body reserves to complete migration and breeding.

Over time, the marked difference in mortality between the sexes may have resulted in large‐scale changes of the population sex ratio with a gradual increase in the proportion of males from 32.2% in 1979 to 61.7% in 2005 (Lehikoinen et al., 2008). Through spring 2013 and 2014, counts of eiders migrating into the Baltic Sea suggested an even stronger (67%) male bias (Berg, 2014). Lehikoinen et al. (2008) suggested that the current decline of the flyway population was intimately linked to large‐scale changes in the population sex ratio, as the two processes occurred in parallel. We found that under the assumption of constant survival of adult males, the skewed adult sex ratio can be explained solely by the increased rate of mortality among nesting females. This is also supported by studies reporting equal proportions of the sexes at hatch (Blums & Mednis, 1996; Swennen, Duiven, & Reyrink, 1979).

A highly skewed sex ratio is, however, not the only driver of structural change in this population. The declining proportion of first‐year birds in The Danish hunting record suggested either a decline in fecundity or first‐year survival (Ekroos, Fox, et al., 2012). Assuming that fecundity has remained stable, our models suggest that first‐year survival from hatching has fallen considerably, that is, from 0.12 to 0.09, between periods of opposing population trends. Observations indicate that an increasing proportion of newly hatched ducklings disappear between the colony at Christiansø in the Baltic Sea and their nursery feeding grounds 18 km away near Bornholm (Kofod & Buchmann, 2016). First‐year survival probability as low as 0.01–0.05 reported from the Gulf of Finland (Hollmen, Lehtonen, Sankari, Soveri, & Hario, 1999) confirms a recent and very dramatic structural change operating at an early life stage. Such a massive die‐off of ducklings might be caused by starvation or increased predation susceptibility as a potential result of high infection rates with acanthocephalan parasites (Hollmen et al., 1999), or by thiamine deficiency (Morner et al., 2017) causing a lower weight gain or neurological disorder in ducklings. Although a decline in adult female survival is likely to have the highest negative impact on the population, low duckling survival also contributes to further reductions in flyway population size.

### 4.2 Harvest management

Harvest rates assessed from independent data sources, that is, ring recoveries and hunting statistics, both indicated a major reduction over time in the contribution of harvest to the mortality of adult females. This is partly due to fewer active, Danish seaduck hunters (Asferg, Bregnballe, Christensen, Clausager, & Clausen, 2003; Christensen, 2005), but also to a set of restrictions on the length of the hunting season on females (Christensen & Hounisen, 2014). The effectiveness of shortening the hunting season depends on both the spatiotemporal distribution of the hunted population and the motivation for hunters to maintain their annual harvest (Sunde & Asferg, 2014). In this case, hunters did not compensate for the shorter season by intensifying their kill of females, in contrast to males (Christensen & Hounisen, 2014). Despite high killrates from hunting throughout the 1970s and 1980s, extremely low natural mortality ensured population growth at that time. Although hunting in this period was intense, our results show that survival of adult females that mainly winter in an entirely hunting‐free area in the Dutch Wadden Sea was only 1% point higher compared to birds subject to hunting in Danish waters. In contrast to the period of population increase, the period of decline following year 2000 was characterized by high natural mortality and low hunting mortality.

### 4.3 Data and knowledge gaps

We found that the estimate of mortality due to hunting was substantially lower when inferred from the ring‐recovery model compared to the hunting statistics, although hunting mortality between 2011 and 2014 was so low that the difference was negligible. While this bias highlights the need for caution and for using independent data for validation of model results, it might also have interesting, underlying causes. For instance, (a) the size of the flyway population might be larger than current estimates suggest, (b) The Danish Wing Survey might not be representative, (c) colonies where birds were ringed might be subject to less hunting than average, (d) *α*_h_ estimates from the multi‐event model might be biased low if the recovery probability from hunting was estimated too high, or (e) low temporal variability of *α*_h_ toward the end of the time series might constitute a risk of bias in either *α*_h_ itself or the cause‐specific recovery probability *r*_h_ (Schaub, 2009; Schaub & Lebreton, 2004; Schaub & Pradel, 2004). Clearly, better monitoring (e.g., regular coordinated and standardized midwinter surveys for more accurate population size estimates and sustained mark–recapture studies involving adult females, ducklings, and ideally adult males) would reduce such potential sources of bias. Estimation of demographic parameters could be further improved by also including birds ringed outside the study colonies.

### 4.4 Recommendations

Sustainable harvest management should aim to protect not only adult females but also first‐winter birds. In fact, one potentially unintended consequence of the female hunting ban implemented in 2014 was a marked reduction in the bag of first‐winter birds, which in the field resemble adult females (Christensen & Hounisen, 2014). Within the European Union, the Annex II of the EU Birds Directive (79/409/EEC) aims to control levels of hunting by listing species that can be legitimately hunted in some (Annex II/B) or all member states (Annex II/A), but the Directive does not regulate national harvest rates. Although common in North America (Klimstra & Padding, 2012), a coordinated flyway approach to harvest management has so far only been applied to one European quarry species, the pink‐footed goose *Anser brachyrhynchus*, where adaptive harvest management has been applied in an attempt to reach a specific target spring flyway population size based on scientific principles (Madsen, Clausen, Christensen, & Johnson, 2016). Given the high, recent rates of natural mortality among females and first‐year individuals, our predictive population projections clearly demonstrate the current need to maintain the hunting ban on female eiders. There is also a pressing need to study the underlying causes of high natural mortality, especially among the nesting female eiders, to target conservation efforts. We believe that investigations of important causes of natural mortality should try to assess the impact of avian and mammalian predation, food limitations causing starvation over the breeding season, and frequency of epidemic disease. In addition, focus should also be directed toward survival of hatchlings during the first months of life because high mortality among hatchlings may later contribute to further population declines due to the lack of recruits. Finally, we advocate that sustainable harvest management of the Baltic/Wadden Sea flyway population of Common Eiders should be founded on the principles of adaptive management, based on continuous monitoring of reproductive success, vital rates, and levels of natural and hunting mortality as crucial components for future evaluations of harvest management.

## CONFLICT OF INTEREST

None declared.

## AUTHOR CONTRIBUTIONS

RST and MF conceived the study. RST developed the statistical and population models, with input from RC, RP, and MF; collated the mark–recapture–recovery data from numerous contributors; analyzed the data; and led the writing of the manuscript. TKC provided Danish hunting data. All authors contributed critically to the drafts and gave final approval for publication.

## Data Availability

Data are available from the Dryad Digital Repository: https://doi.org/10.5061/dryad.97h25b0 (Tjørnløv et al., 2019).

## APPENDIX 1

### CAPTURE–RECAPTURE–RECOVERY DATA USED TO ANALYZE FLYWAY VITAL RATES (STEP 1) AND CAUSE‐SPECIFIC MORTALITY (STEP 2)

**Table:**

Data (recaptures and data from the nonhunted population Vlieland not included in step 2) Step 1: analysis: flyway vital rates; data: capture–recapture–recovery; program: Program MARK Step 2: analysis: hunting mortality of adult females; data: capture–recovery; program: E‐SURGE					
Colony	Age–sex class	Years of ringing First–last year	No. of birds ringed (no. recaptured)	No. of recoveries (shot, nonshot)	% recovered
Vlieland	Ad females	1974–2003	4,223 (733)	1,111	26.3%
Mandø	–	1971–1980	353 (348)	69 (40, 29)	19.5%
Stavns Fjord	–	1970–2007	4,023 (1,680)	917 (483, 434)	22.8%
Hindsholm	–	1989–2014	1,814 (607)	129 (30, 99)	7.1%
Helleholm	–	1982–2015	1,706 (914)	170 (84, 86)	10.0%
Næbbet	–	1982–1996	873 (440)	61 (31, 30)	7.0%
Saltholm	–	1993–2008	1,379 (300)	77 (39, 38)	5.6%
Utklippan	–	1984–2004	531 (327)	54 (10, 44)	10.2%
Christiansø	–	1973–2015	5,759 (2,735)	1,101 (384, 717)	19.1%
Tvärminne	–	1996–2014	1,659 (662)	171 (16, 155)	10.3%
Step 1: Age‐ and sex‐specific survival					
Vlieland	Ad males	1974–1996	1,335 (22)	430	32.2%
Christiansø	Ducklings	1973–1982	5,685 (129)	443	7.8%

### STEP 1—ESTIMATING FLYWAY VITAL RATES

We used the Burnham model, which is particularly suited for quarry species with large numbers of recoveries. The Burnham model provides an analytical framework built into Program MARK that accommodates for combinations of both live and dead encounters. All sets of parameters, survival (*S*), capture probability (*p*), recovery rate (*r*), and site fidelity (*F*), were structured to accommodate for a combined analysis of all colonies with time‐dependent survival and capture probability and different constraints on recovery and site fidelity rates resulting in a subset of candidate models:

**Table:**

Model	AICc	Delta AICc	AICc weights	Model likelihood	Num. Par	Deviance
{*S*(*g***t*) p(*g***t*) *r*(*T*, VL) *F*(*g*)}	123,539.7	0	1	1	433	25,324.561
{*S*(*g***t*) *p*(*g***t*) *r*(*T*) *F*(*g*)}	123,626.2	86.5176	0	0	432	25,413.113
{*S*(*g***t*) *p*(*g***t*) *r*(*T*) *F*(.,VL)}	123,726.1	186.4298	0	0	423	25,531.403
{*S*(*g***t*) *p*(*g***t*) *r*(*T*) *F*(.)}	123,776.4	236.6384	0	0	422	25,583.658
{*S*(*g***t*) *p*(*g***t*) *r*(.) *F*(.,VL)}	123,963.4	423.6583	0	0	422	25,770.678
{*S*(*g***t*) *p*(*g***t*) *r*(.) *F*(*g*)}	124,242.4	702.6952	0	0	431	26,031.334
{*S*(*g***t*) *p*(*g***t*) *r*(.) *F*(.)}	124,393.4	853.657	0	0	421	26,202.721

where *g***t* is the interaction between group (*g*), that is, colony, and time (*t*); *T* is a trend over time; and VL is the abbreviation for the colony of Vlieland.

We speculated that systematic beached bird surveys in The Dutch Wadden Sea would result in a higher recovery rate for the colony of Vlieland relative to the others. Therefore, we also fitted a model with a separate recovery rate *r*(*T*, VL) for birds ringed at Vlieland.

Estimates of adult female survival from the best model, that is, *S*(*g***t*) *p*(*g***t*) *r*(*T*, VL) *F*(*g*).

#### ESTIMATES OF ADULT MALE AND IMMATURE SURVIVAL

Adult male survival from Vlieland and first‐ and second‐year survival from Christiansø were analyzed within single‐site mark–recapture–recovery models (step 1). Adult males and ducklings were combined with the adult females as additional groups and survival parameters of first‐year and second‐year individuals were allowed to differ from survival of the adult females (and from each other), while recapture probabilities *p* of first‐year, second‐year, and third‐year females were allowed to differ from the recapture probability of adult females (and each other). Recovery (*r*) and site fidelity (*F*) rates were allowed to differ between first‐year individuals and the remaining age classes. Survival (*S*), recapture (*p*), and recovery rates (*r*) were always set constant for males and immatures, whereas survival of adult females was set time‐dependent and colony‐specific. Recovery rates of adult females were set common across colonies to facilitate estimation of all parameters.

**Table:**

Estimates and assumptions for the (postbreeding) basic matrix population model			
Parameter	Estimate	Assumption	Reference
Flyway pop size 1970	816,446	Pop size of 1.7mio birds in 1991 and 3.46% growth before.	Joensen (1974)
*S*_0_, first‐year survival	0.12	Representative of the flyway pop	Christiansø data
*S*_1_, second‐year survival	0.83	Representative of the flyway pop	Christiansø data
*S*_ad female_, ad female survival	0.90	Representative of the flyway pop	Mean of 10 study sites
*S*_ad male_, ad male survival	0.92	Representative of the flyway pop	Vlieland data
Fecundity	4.1	Representative of the flyway pop	Christensen and Hounisen (2014)
*σ*, sex ratio at birth	0.50	Equal sex ratio at birth	Lehikoinen et al. (2008)
*b*_2_, breeding propensity of second‐year birds	0	No birds breed before the third year of age	
*b*_3_, breeding propensity of adults	0.70	Estimated by trial and error given the assumptions above	

**Table:**

Estimates and assumptions for the midwinter matrix population model			
Parameter	Estimate	Assumption	Reference
Flyway pop size 1970	816,446	Pop size of 1.7mio birds in 1991 and 3.46% growth before	Joensen (1974)
SW_1_, first‐ to second‐winter survival	0.83	Equals second‐ to third‐year survival between breeding seasons	This study, Christiansø data
SW_2_, second‐ to third‐winter survival	Simulations based on a time series	Equals survival over half a year as second‐year old and over half a year as adult	This study
SW_adF_, adult female winter survival	Simulations based on a time series	Adult female survival is the same between breeding seasons as between winters	This study
SW_adM_, adult male winter survival	0.92 or a similar fall as for the females on the logit scale	Representative of the flyway pop	This study, Vlieland data
Fecundity	Simulations based on a time series	Representative of the flyway pop	Bregnballe (1991)
*b*_2_, breeding propensity of second‐year birds	0	No birds breed before the third year of age	
*b*_3_, breeding propensity of adults	0.70	Estimated based on the other assumptions by trial and error	

### STEP 2—HUNTING MORTALITY OF ADULT FEMALES

Specification of the multi‐event model implemented in program E‐SURGE.

$$\begin{array}{c} 1\\ 2\\ 3\\ 4 \end{array}\left[\begin{array}{c} \begin{array}{ccc} S & * & - \end{array}\\ \begin{array}{ccc} - & - & * \end{array}\\ \begin{array}{ccc} - & - & * \end{array}\\ \begin{array}{ccc} - & - & * \end{array} \end{array}\right],\;\begin{array}{c} 1\\ 2\\ 3 \end{array}\left[\begin{array}{c} \begin{array}{ccc} {}* & - & \begin{array}{cc} - & - \end{array} \end{array}\\ \begin{array}{cccc} - & \alpha_{h} & * & - \end{array}\\ \begin{array}{ccc} - & - & \begin{array}{cc} - & * \end{array} \end{array} \end{array}\right],\;\begin{array}{c} 1\\ 2\\ 3\\ 4 \end{array}\left[\begin{array}{ccc} \begin{array}{c} {}*\\ {}*\\ {}*\\ {}* \end{array} & \begin{array}{c} -\\ r_{h}\\ -\\ - \end{array} & \begin{array}{c} -\\ -\\ \mathrm{ro}\\ - \end{array} \end{array}\right]\;\begin{array}{c} 1\\ 2\\ 3 \end{array}\left[\begin{array}{cccc} \begin{array}{c} {}*\\ -\\ - \end{array} & \begin{array}{c} -\\ \delta_{h}\\ - \end{array} & \begin{array}{c} -\\ -\\ \delta_{o} \end{array} & \begin{array}{c} -\\ {}*\\ {}* \end{array} \end{array}\right]$$

Matrices from left to right: (a) state transitions for survival (*S*); (b) state transitions for the cause of death (*α*_h_, *α*_o_); (c) event process (observation), that is, recovery of a newly dead animal due to hunting or other causes (*r*_h_, *r*_o_); and (d) assignment probability, that is, that an individual dead from either cause is assigned to the correct cause (*δ*_h_, *δ*_o_).

Between occasion *i* and occasion *i +* 1, individuals alive at occasion *i* can either survive with probability *S* or die with the complement probability 1 − *S*, denoted *. Newly dead birds can die due to either hunting or other causes, that is, states 2 and 3, respectively. Hereafter, dead birds move to an old dead state (state 4). Conditional on the states, the event processes (observation) facilitate unbiased estimation of proportions dying due to either cause by correcting for potential differences in cause‐specific recovery rates. Results from the multi‐event model are represented in the main text.

The following people should be acknowledged for their valuable contribution of data and useful advice regarding specific eider colonies:

Henk van der Jeugd, Bruno Ens, and Peter de Boer—data and advice regarding Vlieland.
Henning Noer—data and advice regarding Stavns Fjord and Mandø.
Lars Hansen—data and advice regarding Lillestrand.
Markus Öst, Mikael Kilpi, Kim Jaatinen and Patrik Karell—data and advice regarding Tvärminne.
Peter Lyngs—data and advice regarding Christiansø.
Rolf Larsson—data and advice regarding Utklippan.
Søren K. Pedersen and Benth M. Lange—data and advice regarding Helleholm/Næbbet.
Thomas Kjær Christensen—data and advice regarding Saltholm.
